# 

**Published:** 1806-05

**Authors:** 


### LIST OF NEW MEDICAL PUBLICATIONS.

The Vaccine Contest; or, "Mild Humanity, Reason, Religion, and Truth, against fierce unfeeling Ferocity, overbearing Insolence, mortified Pride, false Faith, and Desperation;" being an exact Outline of the Arguments and interesting Facts adduced by the principal Combatants on both Sides, respecting Cow-pox Inoculation; including a late official Report on this Subject, by the Medical Council of the Royal Jennerian Society. By William Blair, 2s. 6d.

A Treatise on Epilepsy, and the Use of the Viscus Quercinus, or Misletoe of the Oak, in the Cure of that Disease. By H. Frazier, M. D. &c. 2s. 6d. 8vo. sewed.

Surgical Observations on Health. By Mr. Abernethy. Part 2d. 6s. 8vo. bds.

The Medical Observer; containing an impartial Account of advertised Nostrums, their Composition, and the dangerous Consequences that must inevitably arise from their indiscriminate Use, particularly such as are advertised under fictitious Names. To which are added, a few Specimens of the profound Erudition of a Member of this learned Body (being an original Correspondence); with Observations addressed to the Right Honourables, the Right Reverends, and Commoners, who have permitted their Names to appear in the public Prints, in Support of a Practice, which is calculated to destroy many thousand Members of the British Community annually. 2s. 6d.

Observations on Abortion; containing an Account of the Manner in which it is accomplished, the Causes which produced it, and the Method of preventing or treating it. By John Burns. 4s. 6d. 8vo. bds.

---

### TO CORRESPONDENTS.

Communications are received from Dr. Bins, Dr. Wood, Mr. Blegborough, Mr. Dashwood, Dr. Walker, Mr. Young, Dr. Kinglake, and Dr. Bellamy.

